# *Transmembrane Protease Serine 11B* Modulates Lactate Transport Through *SLC16A1* in Pancreatic Ductal Adenocarcinoma—A Functional Link to Phenotype Heterogeneity

**DOI:** 10.3390/ijms26115398

**Published:** 2025-06-04

**Authors:** Dinara Baiskhanova, Maike Menzel, Claudia Geismann, Christoph Röcken, Eric Beitz, Susanne Sebens, Anna Trauzold, Heiner Schäfer

**Affiliations:** 1 Institute of Experimental Cancer Research, UKSH Campus Kiel, Arnold-Heller-Str. 3, Bldg. U30, 24105 Kiel, Germany; stu241794@mail.uni-kiel.de (D.B.); atrauzold@email.uni-kiel.de (A.T.); 2 Department of Pharmacy, Christian-Albrechts-University Kiel, 24118 Kiel, Germany; mmenzel@pharmazie.uni-kiel.de (M.M.); beitz@pharmazie.uni-kiel.de (E.B.); 3 Department of Otorhinolaryngology, Carl-von-Ossietzky University Oldenburg, Ammerländer Heerstraße 114-118, 26129 Oldenburg, Germany; claudia.geismann@uni-oldenburg.de; 4 Department of Pathology, Christian-Albrechts-University Kiel, Arnold-Heller-Str. 3, Bldg. U33, 24105 Kiel, Germany; christoph.roecken@uksh.de; 5 TriBanK, University Hospital Schleswig-Holstein, Campus Kiel, Arnold-Heller-Str. 3, Bldg. U30, 24105 Kiel, Germany; susanne.sebens@email.uni-kiel.de

**Keywords:** carcinogenesis, reverse Warburg metabolism, proteolysis, chaperone

## Abstract

Tumor cell heterogeneity, e.g., in stroma-rich pancreatic ductal adenocarcinoma (PDAC), includes a differential metabolism of lactate. While being secreted as waste product by most cancer cells characterized by the glycolytic Warburg metabolism, it is utilized by a subset of highly malignant cancer cells running the reverse Warburg metabolism. Key drivers of lactate transport are the carrier proteins SLC16A1 (import/export) and SLC16A3 (export). Expression and function of both carriers are controlled by the chaperone Basigin (BSG), which itself is functionally controlled by the transmembrane protease serine 11B (TMPRSS11B). In this study we explored the impact of TMPRSS11B on the phenotype of PDAC cells under reverse Warburg conditions. Amongst a panel of PDAC cell lines, Panc1 and BxPc3 cells were identified to express TMPRSS11B at a high level, whilst other cell lines such as T3M4 did not. ShRNA-mediated TMPRSS11B knock-down in Panc1 and BxPc3 cells enhanced lactate import through SLC16A1, as shown by GFP/iLACCO1 lactate uptake assay, whereas TMPRSS1B overexpression in T3M4 dampened SLC16A1-driven lactate uptake. Moreover, knock-down and overexpression of TMPRSS11B differentially impacted proliferation and chemoresistance under reverse Warburg conditions in Panc1 or BxPc3 and T3M4 cells, respectively, as well as their stemness properties indicated by altered colony formation rates and expression of the stem cell markers Nanog, Sox2, KLF4 and Oct4. These effects of TMPRSS11B depended on both SLC16A1 and BSG as shown by gene silencing. Immunohistochemical analysis revealed a reciprocal expression of TMPRSS11B and BSG together with SLC16A1 in some areas of tumor tissues from PDAC patients. Those regions exhibiting low or no TMPRSS11B expression but concomitant high expression of SLC16A1 and BSG revealed greater amounts of KLF4. In contrast, other tumor areas exhibiting high expression of TMPRSS11B together with BSG and SLC16A1 were largely negative for KLF4 expression. Thus, the differential expression of TMPRSS11B adds to metabolic heterogeneity in PDAC and its absence supports the reverse Warburg metabolism in PDAC cells by the enhancement of BSG-supported lactate uptake through SLC16A1 and subsequent phenotype alterations towards greater stemness.

## 1. Introduction

The exchange of nutrients and metabolites between different cancer cells or between cancer and stromal cells, e.g., fibroblasts, greatly contributes to tumor heterogeneity and malignancy [1,2,3]. In addition to the uptake of glucose and glutamine, which is a hallmark of highly proliferative tumor cells regardless of the oxygen supply (Warburg effect), the differential usage of metabolites like pyruvate and lactate adds to the Warburg metabolism on the one hand [4,5]. On the other hand, the disposal or uptake of these metabolites creates the prerequisite of additional phenotypes and malignant traits. An example is the energetic utilization of lactate released from highly glycolytic Warburg cells by a subset of cancer cells, termed *reverse* Warburg metabolism [6,7,8,9,10]. Uptake and release of lactate and pyruvate are accomplished by members of the solute carrier 16 family, SLC16 [11,12]. SLC16A1 (classically named monocarboxylate transporter 1, MCT1) and SLC16A3 (MCT4) are the relevant isoforms that basically carry the full load of monocarboxylate transport in tumor cells [11,12,13,14].

Surface expression and transport activity of both SLC16A1 and SLC16A3 essentially depend on their interaction with the single transmembrane domain chaperone Basigin (BSG) [15,16]. BSG exists in four splicing variants either containing all three extracellular Ig-like domains (BSG1) or lacking the N-terminal Ig-like domain (BSG2). While BSG1 is expressed only in the retina, BSG2 (now named BSG below) is expressed in all other tissues as well as in malignant tumors in which it is often overexpressed [17,18,19]. BSG modulates SLC16 transport on three levels. Firstly, and classically, the intracellular trafficking of SCL16A1/SCL16A3 to the plasma membrane depends on the complexation with BSG via contact in the transmembrane region [16] and the absence or misfolding of BSG prevents the transporter from reaching the plasma membrane altogether. Secondly, recent studies showed that BSG directly and significantly affects the substrate affinity and transport capacity of SLC16A1 [20,21]. Thirdly, the degree of glycosylation of BSG impacts SLC16 monocarboxylate surface expression and transport capacities [21] possibly by spatially modulating the substrate-enriching microenvironment. In a screen of ovarian cancer cells, it was observed that the capacity of SLC16A1-expressing cells to import lactate correlates with the degree of BSG glycosylation mainly in its membrane proximal Ig-I and the vicinal Ig-C2 domain (unpublished results).

Another mode of control of SLC16 activity linked to BSG is the interaction with the *transmembrane protease serine 11B* (TMPRSS11B). It was recently shown [22] that the presence of TMPRSS11B (also named HATL5 [23]) in the BSG-SLC16A3 complex increases the rate of SLC16A3-driven lactate export in lung cancer cells. Whereas in normal lung tissue TMPRSS11B is scarcely expressed and the lactate export activity of SLC16A3 is moderate, in accordance with limited glycolysis, TMPRS11B overexpression in lung cancer cells enhances SLC16A3-driven lactate secretion and thereby promotes the glycolysis needed for deregulated cell growth. It has been further shown that TMPRSS11B cleaves the extracellular part of BSG [22]. Consequently, the TMPRSS11B-dependent removal of the two Ig-like domains of BSG leads to a conformational change in the BSG-SLC16A3 interacting region [21], forcing the export of lactate in these cancer cells [22]. Though not shown yet, it is also tempting to speculate that a similar action of TMPRSS11B on SLC16A1 as a BSG client protein may regulate the function of this lactate carrier, too. Given the fact that SLC16A1, in contrast to SLC16A3, can run lactate transport in both directions and that the Ig-I domain of BSG promotes its import function [20], a decrease in SLC16A1-driven lactate uptake by BSG shedding would be expected whereas the absence of TMPRSS11B would favor the role of SLC16A1 as a lactate importer. Consequently, tumor cells exhibiting reverse Warburg metabolism would be supported by the absence of TMPRSS1B.

Especially pancreatic ductal adenocarcinoma (PDAC), one of the most malignant tumor entities [24], is characterized by an extended stroma [25,26] and extensive metabolic heterogeneity [27,28,29,30] including the enrichment of *reverse* Warburg cancer cells [31,32]. This pronounced tumor heterogeneity is regarded as a fundamental condition for the quite poor outcome of PDAC [33,34,35,36,37]. Thus, the almost incurability of PDAC due to rapid and invasive progression and therapy resistance relies on highly variable cancer cell subpopulations including those exhibiting cancer stemness properties. Given the pivotal role of *reverse* Warburg cells and their highly malignant potential [31,32,38], we explored the impact of TMPRSS11B expression on SLC16A1-mediated lactate import as well as phenotype alterations in PDAC cells resulting from forced lactate utilization [32,38]. It was found that TMPRSS11B negatively interferes with the lactate import by SLC16A1 and the absence of TMPRSS11B favors lactate uptake and phenotype alterations towards greater malignant properties of PDAC cells, particularly by developing chemoresistance and stem cell-like properties. Moreover, reverse Warburg areas in PDAC patient-derived cancer tissues were identified by high SLC16A1 and BSG expression but absence of TMPRSS11B expression.

## 2. Results

### 2.1. Differential TMPRSS11B Expression in PDAC Cells

**Figure 1:**
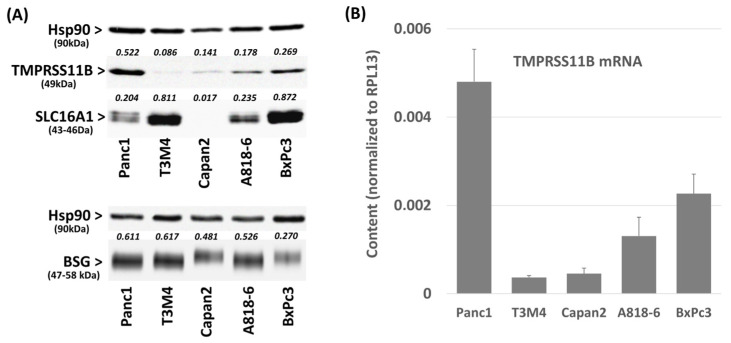
TMPRSS11B, SLC16A1 and BSG expression in PDAC cells. (**A**,**B**), the PDAC lines Panc1, T3M4, Capan2 A818-6 and BxPc3 were analyzed by (**A**) Western blot and (**B**) qPCR for the expression of (**A**,**B**) TMPRSS11B and (**A**) of SLC16A1 and BSG. Western blot data show a representative from three independent experiments together with densitometry analysis using Hsp90 as loading control for normalization (indicated by numbers in italic). The qPCR data represent normalized level against RPL13 as housekeeper and show the mean ± SD from four independent experiments.

Firstly, we analyzed the basal TMPRSS11B expression in the PDAC cell lines Panc1, T3M4, Capan2, A818-6 and BxPc3. As revealed by Western blot analysis (Figure 1A) and qPCR (Figure 1B), Panc1 cells showed the highest basal expression of TMPRSS11B, followed by BxPc3 and A818-6 cells. By contrast, Capan2 and T3M4 cells expressed only small amounts of TMPRSS11B. Furthermore, Panc1 and A818-6 cells expressed SLC16A1 moderately, BxPc3 and T3M4 cells at a high level and Capan2 cells at a very low level (Figure 1A). By contrast, all these cell lines exhibited considerable BSG expression at a comparable level but with variable sizes, indicating variabilities in BSG glycosylation [21] (Figure 1A). Thus, PDAC cells are highly heterogeneous with regard to their expression of TMPRSS11B and SLC16A1.

### 2.2. Effect of TMPRSS11B Expression on SLC16A1-Driven Lactate Uptake in PDAC Vells

**Figure 2:**
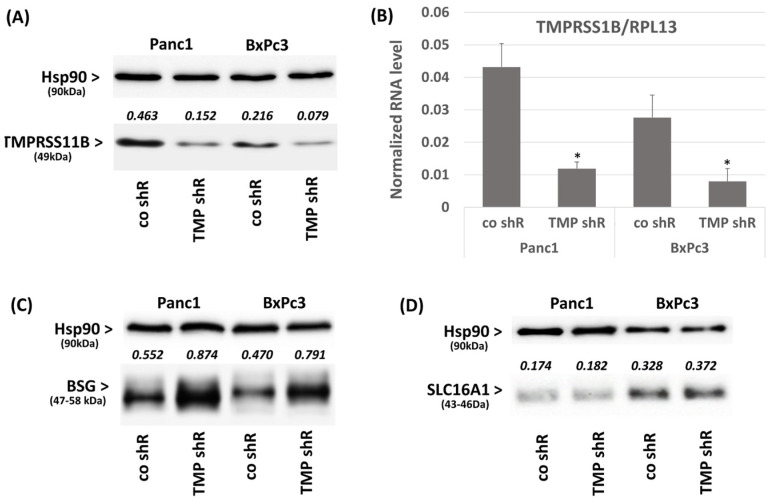

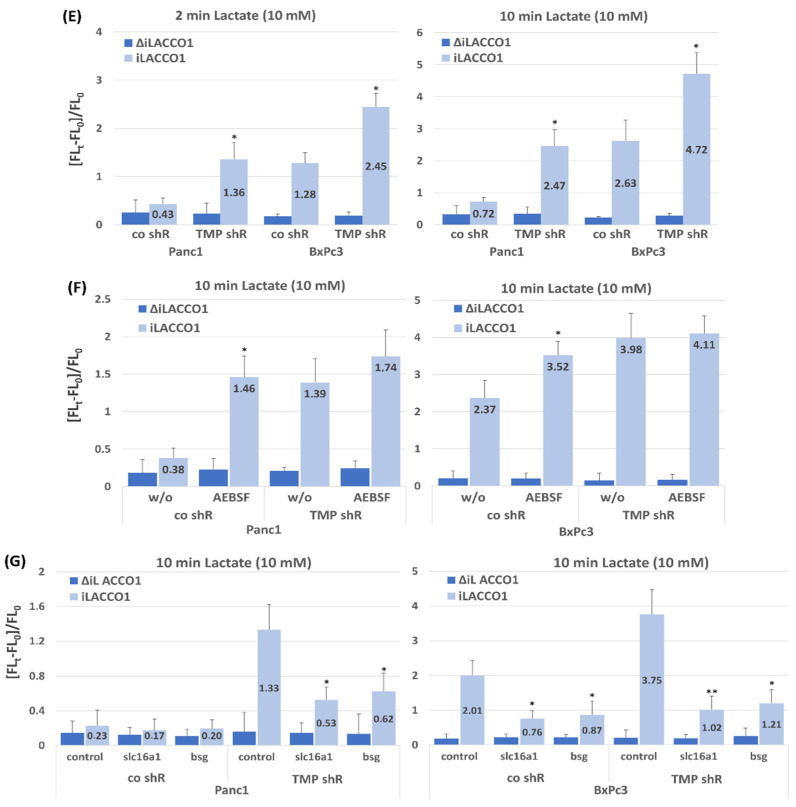
Effect of TMPRSS11B knock-down on SLC16A1 and BSG expression as well as lactate import in Panc1 and BxPc3 cells. The PDAC lines Panc1 and BxPc3 were stably transfected with an expression vector for TMPRSS11B shRNA (TMP shR) or a control shRNA (co shR). Cells were analyzed (**A**) by Western blot or (**B**) qPCR for TMPRSS11B expression or (**C**,**D**) by Western blot for the expression of BSG and SLC16A1. Western blot data show a representative from three independent experiments including the values from densitometry analysis using Hsp90 as loading control for normalization. The qPCR data represent the normalized level against RPL13 as housekeeper and show the mean ± SD from four independent experiments (* *p* < 0.05 compared to co-shRNA). (**E**) To analyze lactate uptake (see also a conventional ^14^C lactate assay for comparison; Supplementary Figure S1), cells transfected with the lactate-specific GFP sensor iLACCO1 or the lactate-insensitive control ΔiLACCO1 were treated with lactate and then recorded for the gain in GFP fluorescence. Data express the Δ*Ft*/*Fo* ratio in cells treated with 10 mM lactate for 2 min or 10 min (mean ± SD from six independent experiments; * *p* < 0.05 compared to co-shR). (**F**) Panc1 and BxPc3 cells stably transfected with TMPRSS11B shRNA (TMP shR) or a control shRNA (co shR) were transfected with ΔiLACCO1 or iLACCO1 for 24 h followed by treatment with 100 µM AEBSF [22] for 16 h or not (w/o) and subsequent lactate uptake measurement. Data express the Δ*Ft*/*Fo* ratio in cells treated with 10 mM lactate for 10 min (mean ± SD from four independent experiments; * *p* < 0.05 compared to “w/o”). (**G**) Panc1 and BxPc3 cells stably transfected with TMPRSS11B shRNA (TMP shR) or a control shRNA (co shR) were treated with control, SLC16A1 (slc16a1) or BSG (bsg) siRNA for 24 h, followed by ΔiLACCO1 or iLACCO1 transfection and lactate uptake measurement 24 h later. Data express the Δ*Ft*/*Fo* ratio in cells treated with 10 mM lactate for 10 min (mean ± SD from four independent experiments; * *p* < 0.05 and ** *p* < 0.01 compared to control siRNA).

Next, it was investigated how the knock-down of TMPRSS11B expression alters BSG and SLC16A1 expression as well as lactate import in PDAC cells. For this purpose, Panc1 and BxPc3 cells showing the highest TMPRSS11B expression were subjected to knock-down of TMPRSS11B by stable shRNA transfection. As shown by Western blot and qPCR, the efficient knock-down of TMPRSS11B (Figure 2A,B) was accompanied by an increase in the BSG protein level whereas SLC16A1 expression did not change (Figure 2C,D). Moreover, TMPRSS11B knock-down resulted in an enhanced uptake of lactate in these PDAC cells as shown by iLACCO1-GFP assay (Figure 2E). Thus, Panc1 cells exhibited the strongest increase in lactate uptake after 2 and 10 min (3.1-fold and 3.4-fold, respectively, higher than in the control siRNA transfected cells) and in BxPc3 cells, a 1.9- and 1.8-fold increase, respectively, was observed. This less pronounced effect of TMPRSS11B knock-down is attributable to the already much greater lactate uptake due to the per se lower TMPRSS11B and higher SLC16A1 expression in BxPc3 cells.

To validate proteolytic activity as the mechanism underlying the effect of TMPRSS11B on lactate uptake, cells were treated with AEBSF, a protease inhibitor shown to block the impact of TMPRSS11B on SLC16A3-driven lactate export [22]. As depicted in Figure 2F, AEBSF treatment led to an enhanced lactate uptake by Panc1 and BxPc3 cells transfected with control shRNA whereas the TMPRSS11B shRNA-transfected cells with an already higher lactate uptake were only marginally affected.

Next, it was tested whether the effect of TMPRSS11B on lactate uptake depends on SLC16A1 and BSG. When silencing SLC16A1 or BSG expression in both shRNA-transfected cell lines by siRNA treatment (Supplemental Figure S2), the increase in lactate uptake under TMPRSS11B knock-down was diminished (Figure 2G). Thus, SLC16A1 silencing decreased lactate uptake in Panc1 and BxPc3 cells from 3.4-fold to 1.4-fold and 1.9- to 1.3-fold, respectively. BSG silencing also resulted in a decrease, but only to 1.8-fold and 1.4-fold, respectively.

**Figure 3:**
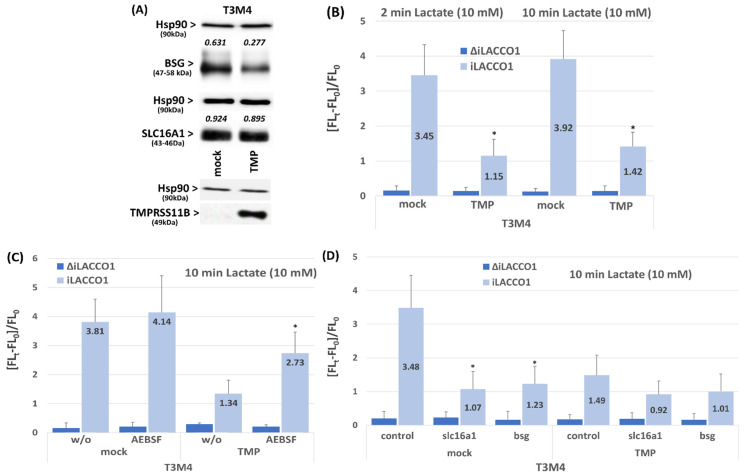
Effect of TMPRSS11B overexpression on SLC16A1 and BSG expression as well as lactate import in T3M4 cells. T3M4 cells were stably transfected with an expression vector for TMPRSS11B cDNA (TMP) or the empty vector (mock). (**A**) Cells were analyzed by Western blot using TMPRSS11B, SLC16A1, BSG and Hsp90 antibodies. A representative result from three independent experiments is shown, including the values (numbers heading the panels for SLC16A1 and BSG) from densitometry analysis using Hsp90 as loading control for normalization. (**B**) Mock- or TMPRSS11B (TMP)-transfected T3M4 cells were transfected with ΔiLACCO1 or iLACCO1 followed by lactate uptake measurement 24 h later. Data express the Δ*Ft*/*Fo* ratio in cells treated with 10 mM lactate for 2 min or 10 min (mean ± SD from four independent experiments; * *p* < 0.05 compared to mock). (**C**) Mock- or TMPRSS11B (TMP)-transfected T3M4 cells were transfected with ΔiLACCO1 or iLACCO1 for 24 h followed by treatment with 100 µM AEBSF [22] for 16 h or not (w/o) and subsequent lactate uptake measurement. Data express the Δ*Ft*/*Fo* ratio in cells treated with 10 mM lactate for 10 min (mean ± SD from four independent experiments; * *p* < 0.05 compared to “w/o”). (**D**) Mock- or TMPRSS11B (TMP)-transfected T3M4 cells were treated with control, SLC16A1 (slc16a1) or BSG (bsg) siRNA for 24 h, followed by ΔiLACCO1 or iLACCO1 transfection and lactate uptake measurement 24 h later. Data express the Δ*Ft*/*Fo* ratio in cells treated with 10 mM lactate for 10 min (mean ± SD from four independent experiments; * *p* < 0.05 compared to mock).

To further support the interconnection between TMPRSS11B and SLC16A1 and BSG, respectively, we studied the effect of TMPRSS11B overexpression on lactate uptake in T3M4 cells expressing TMPRSS11B at a low level (see above). As shown in Figure 3A, overexpression of TMPRSS11B decreased the expression of BSG while SLC16A1 expression remained unaffected. Furthermore, the iLACCO1-GFP assay (Figure 3B) revealed that TMPRSS11B overexpression impaired the profound uptake of lactate by this cell line. Thus, mock-transfected T3M4 cells efficiently imported lactate as monitored after 2 min and 10 min of its addition at 10 mM. By contrast, TMPRSS11B overexpression resulted in a significant decrease in lactate uptake at both time points (by 67% and 63%, respectively). In the presence of AEBSF, the decreasing effect of TMPRSS11B overexpression on lactate uptake in T3M4 cells was abrogated (Figure 3C), again indicating the involvement of proteolytic activity. When SLC16A1 or BSG expression was silenced in T3M4 cells by siRNA treatment (Supplemental Figure S3), lactate uptake was strongly suppressed and the marked difference between mock-transfected and TMPRSS11B-overexpressing cells was not observed anymore (Figure 3D).

### 2.3. Impact of TMPRSS11B Expression on the Cell Cycle of PDAC Cells Under Reverse Warburg Conditions

**Figure 4:**
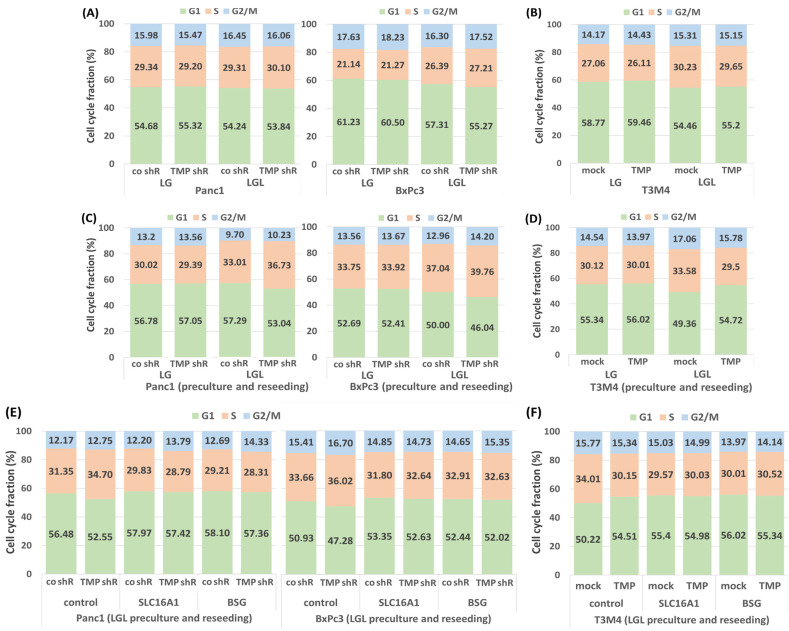
The effect of TMPRSS11B expression on the cell cycle of PDAC cells under reverse Warburg conditions. The PDAC lines Panc1 and BxPc3 transfected with TMPRSS11B (TMP shR) or control (co shR) shRNA and T3M4 cells stably transfected with an expression vector for TMPRSS11B cDNA (TMP) or the empty vector (mock) were cultured in low-glucose (0.5g/L) medium without (LG) or with 20 mM lactate (LGL) for 48 h. Then, cells were either (**A**,**B**) directly submitted to PI staining and cell cycle analysis or (**C**,**D**) reseeded in normal medium (NM) for 24 h followed by PI staining and cell cycle analysis. (**E**) Panc1 and BxPc3 cells transfected with TMPRSS11B shRNA (TMP shR) or control shRNA (co shR) or (**F**) T3M4 cells overexpressing TMPRSS11B (TMP) or not (mock) were first treated with control, SLC16A1 or BSG siRNA for 24 h followed by culture in low-glucose medium plus 20 mM lactate (LGL) for 48 h. Then, cells were reseeded in NM for 24 h followed by PI staining and cell cycle analysis. All data represent the mean from four independent experiments.

Given that the forced lactate uptake under reverse Warburg conditions impacts cell growth [32], it was investigated whether TMPRSS11B expression affects cell cycle progression of the PDAC cells under culture in medium with reduced glucose concentration (0.5 g/L) either without (LG) or with 20 mM lactate (LGL, resembling reverse Warburg conditions). As shown in Figure 4A and Supplementary Table S1A, TMPRSS11B shRNA-transfected Panc1 and BxPc3 cells exhibited no changes in the G1phase and S and G2/M phase fractions under LG culture for 48 h. Under LGL culture (48 h), knock-down of TMPRSS11B led to only little changes in the cell cycle fractions of Panc1 cells whereas in BxPc3 cells, the G1 phase fractions decreased while the S and G2/M phase fractions increased.

By comparison (Figure 4B and Supplementary Table S1B), T3M4 cells, which per se lack considerable TMPRSS11B expression (see above), exhibited a slight increase in the G1 phase fraction and a decrease in the S and G2/M phase fractions under LG or LGL culture when overexpressing TMPRSS11B.

Based on previous findings that the preculture of PDAC cells in LGL results in more pronounced cell cycle alterations after reculture in normal medium (NM) containing 2 g/L glucose [32], we next investigated whether TMPRSS11B expression affects the PDAC cells under these conditions. For this purpose, TMPRSS11B shRNA- or cDNA-transfected and differentially precultured PDAC cells (72 h in LG or LGL) were reseeded and regrown in NM for 24 h. As shown in Figure 4C and Supplementary Table S1C, Panc1 and BxPc3 cells subject to TMPRSS11B knock-down exhibited little changes in the cell cycle fractions if regrown after preculture in LG medium. By comparison, TMPRSS11B knock-down led to significantly lower numbers in the G1 phase and higher numbers in the S and G2/M phase if reseeded from LGL preculture (see Supplementary Table S1C). Thus, the knock-down of TMPRSS11B expression only moderately affects the cell cycle of Panc1 and BxPc3 cells under LG and LGL, but it releases the two PDAC cell lines regrown in NM more efficiently from G1 arrest if the subject of LGL preculture but not LG preculture. Conversely, T3M4 cells reseeded in NM per se exhibited a decreased G1 phase fraction and increased S and G2/M phase fractions upon LGL pretreatment compared to LG pretreatment. This effect by the LGL preculture was prevented by overexpression of TMPRSS11B as indicated by the significantly elevated fraction of the G1 phase and lowered fractions of S and G2/M phases (Figure 4D and Supplementary Table S1D).

These effects of TMPRSS11B knock-down and overexpression on the cell cycle of LGL-pretreated and then NM-regrown Panc1 or BxPc3 cells and T3M4 cells, respectively, were dependent on both SLC16A1 and BSG expression, as shown by siRNA-mediated silencing of SLC16A1 or BSG during LGL culture (Figure 4E,F and Supplementary Table S1E and S1F). Thus, the decrease in the G1 phase fractions as well as the increase in the S and G2/M phases that were seen in the TMPRSS11B shRNA-treated Panc1 and BxPc3 cells were abrogated if SLC16A1 or BSG expression was suppressed by siRNA treatment. Moreover, in comparison to mock cells, TMPRSS11B-overexpressing T3M4 cells showed no difference in cell cycle phase distribution, if SLC16A1 or BSG were knocked down by siRNA.

### 2.4. Impact of TMPRSS11B Expression on the Drug Response of PDAC Cells Under Reverse Warburg Conditions

**Figure 5:**
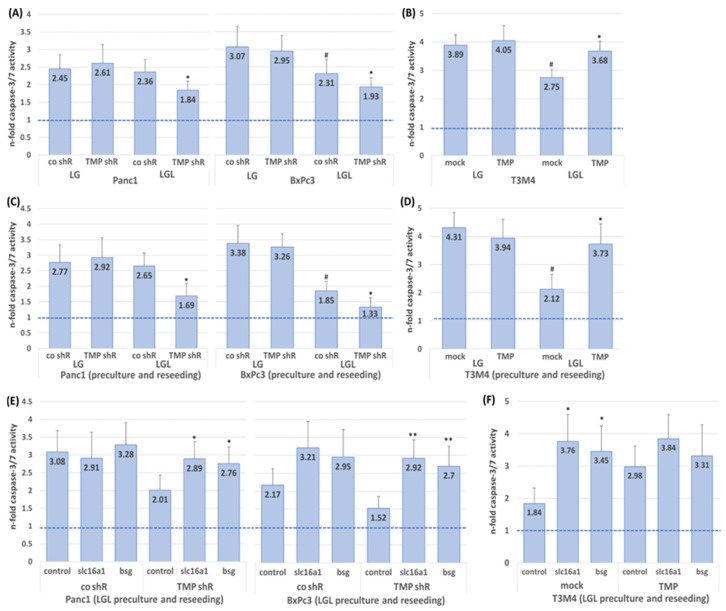
The effect of TMPRSS11B expression on drug response of PDAC cells under reverse Warburg conditions. The PDAC lines (**A**) Panc1 and BxPc3 transfected with TMPRSS11B (TMP shR) or control (co shR) shRNA and (**B**) T3M4 cells stably transfected with an expression vector for TMPRSS11B cDNA (TMP) or the empty vector (mock) were cultured in low-glucose (0.5g/L) medium without (LG) or with 20 mM lactate (LGL) for 48 h. Then, cells were either left untreated or were treated with 10 µg/mL gemcitabine for 30 h and apoptosis was determined by caspase-3/7 assay. (**C**,**D**) PDAC cells were pretreated with LG or LGL for 72 h and then reseeded in normal medium. Then, cells were either left untreated or were treated with 10 µg/mL gemcitabine for 30 h and apoptosis was determined by caspase-3/7 assay. (**E**,**F**) PDAC cells pretreated with control, SLC16A1 or BSG siRNA for 24 h were cultured in LGL for 48 h and then reseeded in normal medium. After 24 h, cells were either left untreated or were treated with 10 µg/mL gemcitabine for 30 h and apoptosis was determined by caspase-3/7 assay. Data are expressed as n-fold of caspase activity in untreated cells and represent the mean ± from at least four independent experiments. Statistical significances are indicated as follows: (**A**,**C**), * *p* < 0.05 compared to “co shR/LGL” and # *p* < 0.05 compared to “co shR/LG”; (**B**,**D**), * *p* < 0.05 compared to “mock/LGL” and # *p* < 0.05 compared to “mock/LG”; (**E**), * *p* < 0.05 and ** *p* < 0.01 compared to “TMP shR/control siRNA”; (**F**), * *p* < 0.05 compared to “mock/control siRNA”.

Next, it was investigated whether TMPRSS11B expression affects the drug response of the PDAC cells under reverse Warburg conditions. For this purpose, PDAC cells were cultured in LG or LGL medium for 48 h followed by treatment with the established anti-cancer drug gemcitabine (10 µg/mL) for 30 h. The analysis of apoptotic cell death by caspase-3/7 assay (Figure 5A) revealed that TMPRSS11B shRNA-transfected Panc1 and BxPc3 cells under LG culture exhibited only small differences in gemcitabine-induced rates of caspase-3/7 activity (2.61- versus 2.45-fold and 2.95- versus 3.07-fold, respectively). By contrast, a decrease in gemcitabine-induced rates of caspase-3/7 activity was noted in TMPRSS11B shRNA-transfected Panc1 cells (1.84- versus 2.36-fold in control shRNA- transfected cells) and BxPc3 cells (1.93- versus 2.31-fold in control shRNA-transfected cells) under LGL culture (48 h). Notably, lower gemcitabine-induced rates of caspase-3/7 activity under LGL compared to LG conditions were already seen in BxPc3 transfected with control shRNA (2.31- in LGL versus 3.07-fold in LG), in line with the per se lower expression level of TMPRSS11B in this cell line (Figure 5A).

Moreover, T3M4 cells (Figure 5B), which per se showed a considerably lower response towards gemcitabine if cultured in LGL (yielding lower gemcitabine-induced rates of caspase-3/7 activity (2.75- in mock/LGL versus 3.89-fold in mock/LG)), responded significantly stronger to gemcitabine treatment if overexpressing TMPRSS11B as indicated by the higher rates of caspase-3/7 activity (3.68- in TMP/LGL versus 2.75-fold in mock/LGL).

Based on previous findings that preculture of PDAC cells in LGL reduces responsiveness towards gemcitabine when recultured in NM [32], we next investigated whether TMPRSS11B expression affects the drug response of the PDAC cells under these conditions. For this purpose, TMPRSS11B shRNA- or cDNA-transfected and differentially precultured PDAC cells (72 h in LG or LGL) were reseeded and regrown in NM for 24 h followed by gemcitabine treatment for 30 h. As shown in Figure 5C, Panc1 and BxPc3 cells subjected to TMPRSS11B knock-down exhibited significantly altered rates of gemcitabine-induced caspase-3/7 activity if reseeded after LGL but not LG preculture. In LGL-precultured Panc1 and BxPc3 cells, gemcitabine-induced caspase-3/7 activity decreased from 2.65-fold to 1.69-fold and 1.85-fold to 1.33-fold, respectively. Again, BxPc3 cells already exhibited a significantly decreased rate of gemcitabine-induced caspase-3/7 activity by LGL culture alone (1.85-fold versus 3.38-fold in LG).

To demonstrate the SLC16A1 and BSG dependency of the TMPRSS11B knock-down effect on the reduced gemcitabine response under these conditions, Panc1 and BxPc3 cells transfected with control or TMPRSS11B shRNA were treated with control, SLC16A1 or BSG siRNA followed by LGL culture for 72 h and subsequent reseeding in NM. As shown in Figure 5E, the less pronounced rate of gemcitabine-induced caspase-3/7 activity (2.01-fold) in Panc1 cells under TMRSS11B knock-down was abolished by SLC16A1 and BSG siRNA treatment, yielding a 2.89- and 2.76-fold, respectively, induction of caspase-3/7 activity. In BxPc3 cells, the response to gemcitabine (2.17-fold gain in caspase-3/7 activity) was already increased in the control shRNA transfectants by pretreatment with SLC16A1 or BSG siRNA (yielding a 3.21- and 2.95-fold gain in caspase-3/7 activity, respectively). In BxPc3 cells with TMPRSS11B knock-down, the even lower response to gemcitabine (1.52-fold gain in caspase-3/7 activity) was similarly reversed by SLC16A1 or BSG siRNA treatments (yielding a 2.92- and 2.70-fold gain in caspase-3/7 activity, respectively).

In T3M4 cells, the reduced drug response mediated by LGL pretreatment prior to NM reseeding (yielding a 2.1-fold gain in caspase-3/7 activity) compared to LG pretreatment prior to NM reseeding (yielding a 4.31-fold gain in caspase-3/7 activity) was significantly reversed (yielding a 3.73-fold gain in caspase-3/7 activity) by overexpression of TMPRSS11B (Figure 5D). As shown in Figure 5F, the much less pronounced LGL-induced resistance in TMPRSS11B-overexpressing T3M4 cells was only slightly affected by the treatment with SLC16A1 and BSG siRNA, respectively (yielding a 3.84- and 3.31-fold compared to a 2.98-fold gain in caspase-3/7 activity under control siRNA treatment). By contrast, mock-transfected T3M4 cells revealed a strong sensitizing effect by SLC16A1 and BSG siRNA treatment, yielding a 3.76- and 3.45-fold gain in caspase-3/7 activity, respectively, compared to a 1.84-fold gain in caspase-3/7 activity in control siRNA-treated T3M4 cells without TMPRSS11B overexpression.

### 2.5. Impact of TMPRSS11B Expression on Stemness Properties of PDAC Cells Under Reverse Warburg Conditions

**Figure 6:**
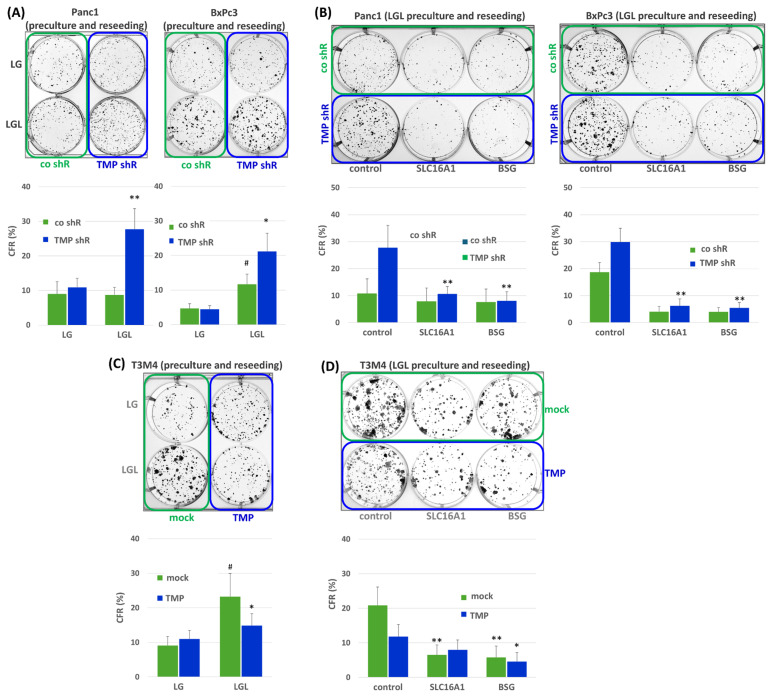
Impact of TMPRSS11B expression on colony formation capacity of PDAC cells under reverse Warburg conditions. The PDAC lines Panc1 and BxPc3 stably transfected with TMPRSS11B (TMP shR) or control (co shR) shRNA were (**A**) precultured in LG or LGL for 72 h and then reseeded in NM or (**B**) pretreated with control, SLC16A1 or BSG siRNA followed by culture in LGL for 48 h and subsequent reseeding in NM. (**C**,**D**) T3M4 cells stably transfected with the TMPRSS11B cDNA (TMP) vector or the empty (mock) vector were (**C**) cultured with LG or LGL for 72 h and then reseeded in NM or (**D**) pretreated with control, SLC16A1 or BSG siRNA followed by culture in LGL for 48 h and subsequent reseeding in NM. Colony formation rate (CFR) was determined after 6–10 d by visualizing colonies by crystal violet staining. CFR is expressed as colonies formed from 500 cells initially seeded (%). Representative images are shown, and histograms represent the mean ± SD from (**A**,**C**) six independent and (**B**,**D**) four independent experiments. Statistical significances are indicated as follows: (**A**), * *p* < 0.05 compared to co-shR and # *p* < 0.05 compared to “co shR/LG”; (**B**), * *p* < 0.05 and ** *p* < 0.01 compared to control siRNA; (**C**), * *p* < 0.05 compared to mock and # *p* < 0.05 compared to “mock/LG”; (**D**), * *p* < 0.05 and ** *p* < 0.01 compared to control siRNA.

Next, it was investigated whether TMPRSS11B affects the stem cell properties of the PDAC cells under reverse Warburg conditions [32]. For this purpose, Panc1 and BxPc3 cells stably transfected with TMRSS11B shRNA were reseeded from LG or LGL preculture (72 h) in NM and analyzed for colony formation after 6-10 d. As shown in Figure 6A, the colony formation rates of TMPRSS11B shRNA-transfected PDAC cells were much greater than those of control shRNA-transfected cells after LGL preculture (Panc1: 27.8 ± 5.9% vs. 8.9 ± 2.6%, BxPc3: 21.1 ± 4.5% vs. 11.7 ± 3.2%) but not after LG preculture (Panc1: 10.9 ± 2.4% vs. 9.1 ± 3.5%, BxPc3: 4.4 ± 0.4% vs. 4.7 ± 0.6%) before reseeding. Thus, TMPRSS11B knock-down greatly favored the colony formation capacity of the PDAC cells that underwent reverse Warburg metabolism before regrowth in normal medium. In the case of control shRNA-transfected BxPc3 cells, LGL preculture already led to enhanced colony formation compared to LG preculture, which is in line with the greater lactate uptake in this cell line per se expressing TMPRSS11B at a lower and SLC16A1 at a higher level (see above). When silencing SLC16A1 or BSG expression by siRNA treatment prior to the LGL preculture, the colony formation rates after NM reseeding revealed no differences anymore between the control or TMPRSS11B shRNA-transfected Panc1 and BxPc3 cells (Figure 6B).

In T3M4 cells, the per se significantly higher colony formation rate after LGL preculture compared to LG preculture and reseeding in NM was abrogated by TMPRSS11B overexpression (Figure 6C). Thus, mock-transfected T3M4 cells exhibited a colony formation rate of 23.3 ± 6.7% (LGL preculture) versus 9.1 ± 2.2% (LG preculture), whereas TMPRSS11B-transfected cells exhibited a colony formation rate of 14.8 ± 3.9% (LGL preculture) versus 11.0 ± 1.8% (LG preculture). Silencing of SLC16A1 or BSG (Figure 6D) in T3M4 mock cells abrogated the greater colony formation rate. Little differences in colony formation were seen in T3M4 cells overexpressing TMPRSS11B along with its inhibitory effect on colony formation.

### 2.6. Impact of TMPRSS11B Expression on Stem Cell Marker and Reprogramming Factor Expression in PDAC Cells Under Reverse Warburg Conditions

**Figure 7:**
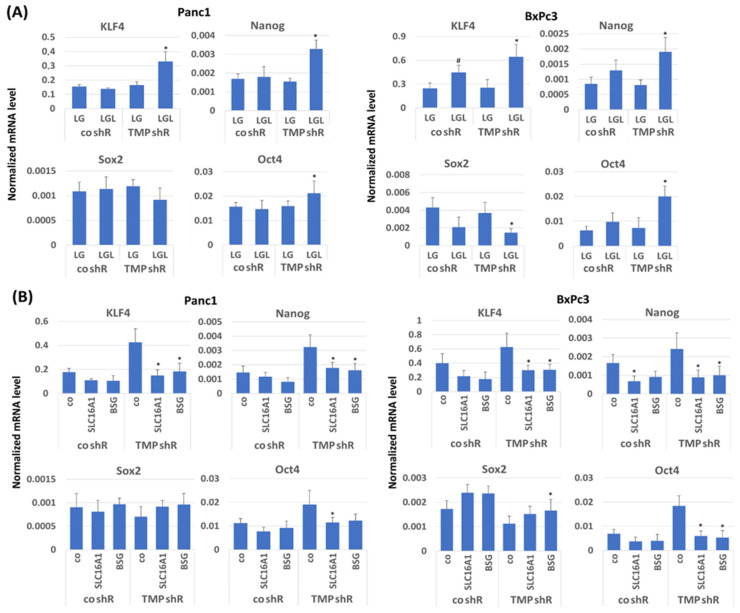

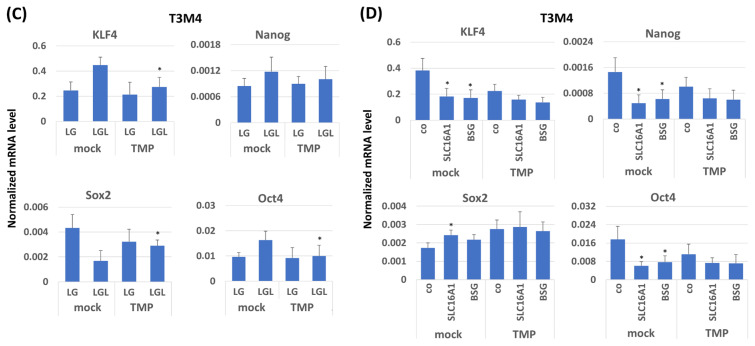
Impact of TMPRSS11B expression on stemness and reprogramming factor expression in PDAC cells under reverse Warburg conditions. The PDAC lines Panc1 and BxPc3 stably transfected with TMPRSS11B (tmprss11b) or control (co) shRNA were (**A**) precultured in LG or LGL for 72 h and then reseeded in NM or (**B**) pretreated with control, SLC16A1 or BSG siRNA followed by culture in LGL for 48 h and then reseeding in NM for 24 h. (**C**,**D**) T3M4 cells stably transfected with the TMPRSS11B cDNA vector or the empty (mock) vector were (**C**) cultured with LG or LGL for 72 h and then reseeded in NM or (**D**) pretreated with control, SLC16A1 or BSG siRNA followed by culture in LGL for 48 h and then by reseeding in NM for 24 h. Afterwards, qPCR was performed to analyze KLF4, Nanog, Oct4 and Sox2 mRNA expression using RPL13 mRNA as housekeeper for normalization. Data are expressed as the normalized mRNA level and show the mean ± SD from six (**A**,**C**) and four (**B**,**D**) independent experiments. Statistical significances are indicated as follows: (**A**), * *p* < 0.05 compared to “co shR/LGL” and # *p* < 0.05 compared to “co shR/LG”; (**B**), * *p* < 0.05 compared to “TMP shR/control siRNA”; (**C**), * *p* < 0.05 compared to “mock/LGL”; (**D**), * *p* < 0.05 compared to “mock/control siRNA”.

To verify and further delineate the role of TMPRSS11B in cancer cell stemness under reverse Warburg conditions, Panc1 and BxPc3 transfected with control or TMPRSS11B shRNA were differentially precultured (72 h in LG or LGL) and then reseeded in NM for 24 h. Stemness marker and reprogramming factor expression were analyzed by qPCR. As shown in Figure 7A, TMPRSS11B knock-down in Panc1 and BxPc3 cells subjected to LGL culture (72 h) before reseeding in NM for 24 h led to significantly elevated levels of KLF4, Nanog and Oct4 compared to LG preculture, while Sox2 expression was slightly decreased. This expression pattern was in line with previous observations on the acquisition of stem cell properties in PDAC cells under reverse Warburg conditions [32]. To confirm the dependency of this effect on SLC16A1 and BSG expression, cells were siRNA treated (24 h) prior to LGL preculture (48 h) and subsequent reseeding in NM. (24 h).

As shown in Figure 7B, both SLC16A1 and BSG knock-down diminished the increasing effect of the TMPRSS11B knock-down on the expression of KLF4, Nanog and Oct4 in Panc1 and BxPc3 cells upon LGL preculture for 72 h and subsequent reseeding in NM. Likewise, Sox2 expression was less diminished by TMPRSS11B knock-down in LGL-pretreated cells, if SLC16A1 or BSG expression had been knocked down. In T3M4 cells, the overexpression of TMPRSS11B diminished the increasing effect of LGL preculture on KLF4, Oct4 and Nanog expression after NM reseeding, while Sox2 expression significantly increased (Figure 7C). The silencing of SLC16A1 or BSG prevented the increase in KLF4, Oct4 and Nanog as well as the decrease in Sox2 in mock-transfected T3M4 cells after LGL preculture and subsequent reseeding in NM (Figure 7D). When overexpressing TMPRSS11B, the decreased response of T3M4 cells to LGL pretreatment in terms of KLF4, Oct4, Nanog and Sox2 expression was not further reduced by SLC16A1 and BSG silencing, confirming the dependency of this TMPRSS11B effect on SLC16A1 and BSG.

### 2.7. KLF4 Expression Colocalizes with SLC16A1/BSG Co-Expressing Regions in Human PDAC Tissue Reciprocally to TMPRSS11B Expression

**Figure 8:**
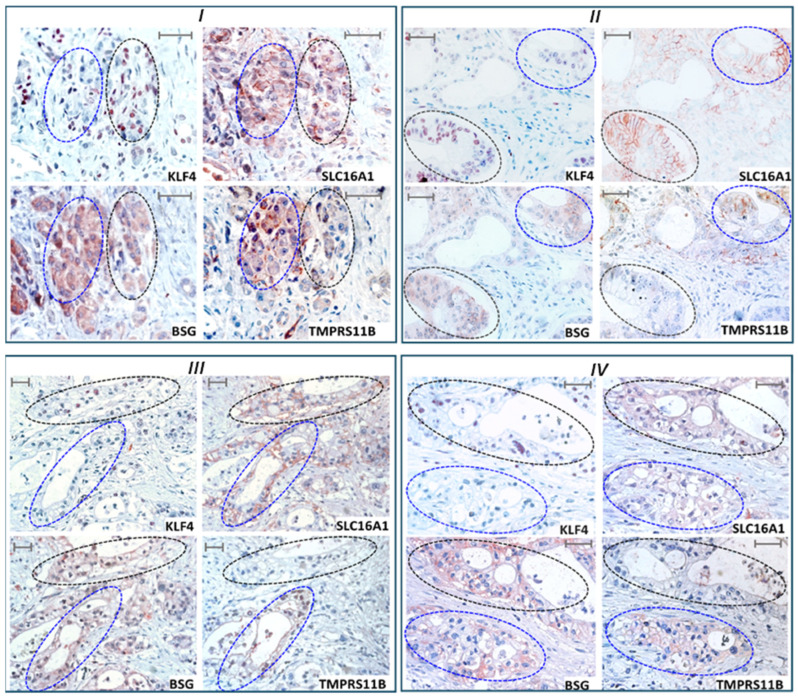
KLF4, SLC16A, BSG and TMPRSS11B expression in human PDAC tissue. Expression of KLF4, SLC16A1, BSG and TMPRSS11B was analyzed by immunohistochemistry in tumor tissues from PDAC patients (all *T3N1M0*). Four different cases (**I**–**IV**) out of a cohort of twenty-one patients are shown displaying representative expression patterns. Neighbored regions with low TMPRSS11B expression are encircled by dotted black lines and with high TMPRSS11B expression are encircled by dotted blue lines. The scale bars indicate 50 µm.

To validate a connection between TMPRSS11B-, BSG- and SLC16A1-driven cancer stemness clinically, tumor specimens from PDAC patients were analyzed by immunohistochemistry (Figure 8). From a total cohort of 31 patients (all T3N1M0 and of grade 2-4), all three targets were detectable in 21 cases. In eight cases, no TMPRSS11B was detectable, while BSG and SLC16A1 were not detectable in two and three cases, respectively.

In the 21 cases in which all three targets were detectable, SLC16A1 and BSG expression was mostly colocalized: from 84 areas analyzed, 69 (=82.1%) exhibited significant costaining for both BSG and SLC16A1. These regions of SLC16A1/BSG colocalization only partially colocalized with TMPRSS11B (Figure 8). In detail, 40 areas (=58.0%) were rather negative for TMPRSS11B expression (scoring <1) and 29 areas (=42.0%) were positive for TMPRSS11B expression (scoring 1–2).

**Figure 9:**
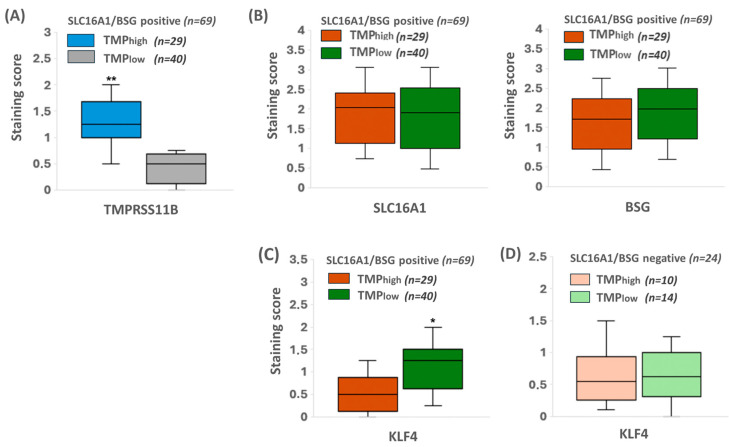
KLF4 expression correlates with high SLC16A1 and BSG expression in human PDAC tissue reciprocal to TMPRSS11B expression. From consecutive immunohistochemically stained specimens (*n* = 21), four representative microscopic fields (in total *n* = 84) were evaluated using the IHC scoring (see 2.12). Boxes cover the 0.25 and 0.75 quantiles and whisker length the medial deviation, * *p* < 0.05 and ** *p* < 0.01. (**A**) TMPRSS11B expression in the 69 microscopic fields with BSG/SLC16A1 co-expression at a high (29/69 at score >1) and low (40/69 at score <1) level. (**B**) BSG and SLC16A1 expression in TMPRSS11B high versus TMPRSS11B low tumor regions. (**C**) Nuclear KLF4 expression in the SLC16A1/BSG co-expressing regions either in the presence or absence of TMPRSS11B. (**D**) Nuclear KLF4 expression in the regions lacking SLC16A1/BSG co-expression (*n* = 24) either in the presence or absence of TMPRSS11B.

To verify the reverse Warburg state along with the stemness characteristics, we explored the expression of the reprogramming factor KLF4 that essentially contributes to PDAC development and cancer stemness [39,40] and has been recently shown to be substantially associated with this metabolic phenotype [32]. In the 69 areas with BSG/SLC16A1 colocalization, nuclear KLF4 expression was detectable at variable degrees (Figure 8 and Figure 9C). Most notably, when comparing BSG/SLC16A1-co-expressing but TMPRSS11B-negative regions (*n* = 40) in close vicinity with BSG/SLC16A1-co-expressing and TMPRSS11B-positive regions (*n* = 29), as depicted in Figure 9A,B, a significantly stronger KLF4 expression was detected in the former (Figure 9C). Thus, TMPRSS11B- and SLC16A1/BSG-co-expressing regions exhibited staining of nuclear KLF4 at a score of <1, whereas SLC16A1/BSG-co-expressing regions with low or no TMPRSS11B expression exhibited nuclear KLF4 staining at a score of >1 (Figure 9C). These TMPRSS11B-associated differences in KLF4 staining were not observed in analyzed regions (*n* = 24; score <1) lacking SLC16A1 and BSG co-expression (Figure 9D), thus underscoring the reciprocal association between TMPRSS11B and BSG/SLC16A1 co-expression with regard to KLF4-expressing reverse Warburg cells with stem cell-like properties.

## 3. Discussion

The flux of monocarboxylates such as pyruvate and lactate are part of metabolic coupling amongst tumor cells as well as between tumor and stroma cells [10,41,42]. Given the fact that the bulk mass of malignant tumor cells is characterized by unleashed proliferation, the predominant metabolic phenotype is aerobic glycolysis, a condition known as the Warburg effect. While yielding only a little net production of energy, high-rate glycolysis serves for the generation of nucleosides for DNA synthesis and substrates for biomass production. An important hallmark of these highly glycolytic tumor cells is the high need for the recovery of NAD^+^ from NADH [43,44], which is managed by the conversion of the glycolytic end-product pyruvate towards lactate by the action of lactate dehydrogenase A (LDHA). Lactate then needs to be transported out of the cell to avoid intracellular acidification and to hold on with the pyruvate conversion. The export of lactate is driven by two members of the SLC16A1 carrier family—SLC16A1 and SLC16A3 [12,45]—assisted by the chaperone BSG [15,16,46]. While SLC16A3 is incapable of running lactate inward transport [14], SLC16A1 can act as both a lactate exporter and importer [20,45]. The cellular modalities underlying the differential control of SLC16A1 and SLC16A3 by BSG as well as the molecular mechanisms are only partially understood.

Notably, the proteolytic cleavage of BSG by the sheddase TMPRSS11B was shown to enforce lactate export by SLC16A3 in lung cancer [22]. In this context, the removal of the Ig-I domain facilitates the export of lactate by SLC16A3. Given the fact that SLC16A1, in contrast to SLC16A3, is capable of running lactate transport in both directions and that the Ig-I domain of BSG is essential in its import function [20], it was hypothesized that TMPRSS11B-mediated BSG cleavage would result in the shutdown of the SLC16A1-driven uptake of lactate but favoring its export. Indeed, overexpression of TMPRSS11B reduced lactate uptake in PDAC cells, whereas the deletion of TMPRSS11B expression strongly enhanced lactate uptake—an effect depending on the expression of both SLC16A1 and BSG. Accordingly, knock-down of either one of both abrogated the impact of TMPRSS11B deficiency on lactate uptake in PDAC cells.

Lactate import is seen in a subset of tumor cells, known as reverse Warburg cells. These highly malignant cancer cells are less glycolytic and characterized by an oxPhos metabolism. Recent studies showed that reverse Warburg metabolism creates a metabolic niche for stem cell-like properties, giving rise to chemoresistant- and tumor relapse-initiating cancer cells [32,36,47,48], which mainly accounts for the poor prognosis of cancer patients associated with tumoral SLC16A1 expression [31,49,50,51,52]. As demonstrated here, TMPRSS11B deficiency in PDAC cells greatly affected the phenotype of PDAC cells, if growing under reverse Warburg conditions implemented by glucose scarcity and exposure to elevated concentrations of lactate. Thus, PDAC cells adopted a more drug-resistant and stem cell-like phenotype (shown by greater colony formation capacity, altered expression level of the reprogramming factors KLF4, Nanog, Oct4 or Sox2 and reduced apoptosis induction after gemcitabine treatment), if grown under reverse Warburg conditions and TMPRSS11B knock-down. This was particularly seen in the PDAC cell line Panc1 exhibiting strong TMPRSS11B expression and per se no reverse Warburg phenotype.

Consequently, tumor cells exhibiting reverse Warburg metabolism would be supported by the absence of TMPRSS1B and thereby the lack of BSG cleavage, as seen in, e.g., T3M4 cells or in Panc1 and BxPc3 cells after knock-down of TMPRSS11B. Overexpression of TMPRSS11B, in turn, diminished lactate uptake in these cells, e.g., in T3M4, and their capacity to adopt the reverse Warburg metabolism as a driver of a malignant phenotype. This scenario may explain the contrary findings on TMPRSS11B expression in different tumor entities. In contrast to lung and gastric cancer [22,53,54] exhibiting TMPRSS11B overexpression, oral, cervical, esophageal and head and neck carcinomas lack considerable TMPRSS11B expression [23,55]. Thus, in TMPRSS11B-expressing tumors, the mass of Warburg cells prevails and determines the malignant phenotype, while in TMPRSS11B-negative tumors, reverse Warburg cells are of greater importance. Being essential for the heterogeneity of many malignant tumors, these reverse Warburg cancer cells might differentially contribute to malignant progression [56] depending on the presence of SLC16A1 and BSG as well as the absence of TMPRSS11B.

At sufficient glucose supply, either under aerobic or anaerobic conditions, TMPRSS11B favors glycolysis along with the greater capacity of lactate disposal through both SLC16A1 and SLC16A3 [12,22]. Thus, proteolytic cleavage of the Ig-C2/Ig-I domain of BSG may lead to an altered conformation of the BSG/SLC16A3 and BSG/SLC16A1 conformation, supporting a forced export of lactate as a prerequisite for enhanced glycolysis and cancer cell growth [22]. This is supported by recent findings on SLC16A3 [22] and SLC16A1 (Supplementary Figure S4). Since SLC16A3 prevails in lactate export in Warburg tumors, the action of SLC16A1 in this context is largely additive. By contrast, under glucose shortage, uptake of lactate provides an essential advantage for those cancer cells expressing SLC16A1, which, in contrast to SLC16A3, also drives the import of lactate. This function of SLC16A1 requires the integrity of the Ig-I domain of BSG [20] that is therefore suppressed by TMPRSS11B-mediated BSG cleavage. Thus, SLC16A1-expressing cancer cells, e.g., in PDAC, acquire a reverse Warburg metabolism preferentially in the absence of TMPRSS11B expression, as supported by our experimental data.

As further shown for patient-derived PDAC tissues, some tumoral areas reveal considerable TMPRSS11B expression with only partial colocalization with SLC16A1 and BSG (Figure 8 and Figure 9B). By contrast, several tissue areas with high SLC16A1 and BSG expression lack co-expression with TMPRSS11B. It can be envisioned that, besides SLC16A1/BSG-TMPRSS11B-co-expressing cells with Warburg phenotype, those cancer cells expressing SLC16A1/BSG but lacking TMPRSS1B (Figure 9B) behave as reverse Warburg cells enriched in the progressed cancer stages investigated here. This is underscored by the fact that in SLC16A1/BSG-expressing but TMPRSS11B-deficient cancer cells, the nuclear expression levels of reprogramming factors such as KLF4 are elevated (Figure 9C), a condition identified as a hallmark of reverse Warburg cells [32,47]. Thus, TMPRSS11B expression is reciprocal to the highly malignant reverse Warburg cells expressing SLC16A1 and BSG, giving rise to relapse-forming and also metastasizing cancers [57,58]. However, more comprehensive studies are needed correlating the clinical outcome of PDAC patients with TMPRSS11B expression and to understand the role of TMPRSS11B as a prognostic marker in this tumor entity, as already performed in other cancer entities but with contrary findings. Thus, in lung and gastric cancer, high TMPRSS11B expression along with Warburg metabolism and forced tumor growth associates with an unfavorable prognosis [22,53,54], whereas in oral, cervical, esophageal and head and neck cancers, it is the lack of TMPRSS11B expression [23,55]. It can be envisioned that the latter cases, such as PDAC, are particularly governed by metabolic heterogeneities resulting in niches enriched for reverse Warburg cells.

Together, in cancer microenvironments, an interdependent system of glucose/pyruvate/lactate energy metabolism is in place that affects the cellular redox status and modulates BSG/SLC16 monocarboxylate transport. Particularly, the differential impact of BSG on the directionality of pyruvate and/or lactate flux in the cancer cells (depending on its structural variations such as glycosylation and TMPRSS11B-mediated cleavage) fosters the growth of either glycolytic Warburg or non-glycolytic reverse Warburg cells. Notably, besides TMPRSS11B, another protease—ADAM12—has been reported to cleave BSG in a similar fashion [59], though its impact on SLC16 activity still needs to be shown. Accordingly, concepts to treat cancer with inhibitors of BSG sheddases like TMPRSS11B [53] or ADAM12 should be considered with caution, since highly malignant reverse Warburg cells would be supported by such treatments.

## 4. Materials and Methods

### 4.1. Reagents and Chemicals

G418 was provided from Capricorn Scientific (Ebsdorfergrund, Germany), Puromycine from Santa Cruz (Heidelberg, Germany) and AEBSF from Merck (Darmstadt, Germany). Cell culture media were purchased from Pan Biotech (Aidenbach, Germany).

### 4.2. Cell Lines and Culture

The human PDAC cell lines Panc1, BxPc3 and Capan2 were purchased from the DSZM (Braunschweig, Germany). T3M4 cells were kindly provided by H. Friess (Heidelberg, Germany) and A818-6 cells were a gift from H. Kalthoff (Kiel, Germany). Culture conditions were as described recently [60,61]. Cell line authenticity was checked by STR-profiling.

### 4.3. RNA Preparation and Real-Time PCR

Isolation of RNA was performed using the Monarach^®^ Total RNA Miniprep Kit (NEB, Frankfurt, Germany) following the manufacturer’s instructions. Reverse transcriptions into single-stranded cDNA and qPCR (Real-Time Thermocycler CFX Connect™, Bio-Rad, Feldkirchen, Germany) were carried out using the SYBR-Green assay (Blue S’ Green qPCR, Biozym, Hamburg, Germany). All primers (Eurofins, Ebensburg, Germany) were used at a final concentration of 0.2 µM. Cycling conditions were as follows: 95 °C 7 min initial denaturation followed by 45 cycles at 95 °C 5 sec/60 °C 30 sec. The following primer sets were used: KLF4 forw/rev; 5′-GGGAGAAGACACTGCGTCAA-3′/5′-GGAAGTCGCTTCA TGTGGGA-3′, Oct4 forw/rev; 5′-GGTGGAGGAAGCTGACAACA-3′/5′-GTTCGCTTT CTCTTTCGGGC-3′, Sox2 forw/rev; 5′-TCCCATCACCCACAGCAAATGA-3′/5′-TTTCTTGTCGGCATCGCGGTTT-3′, Nanog forw/rev; 5′-ACATGCAACCTGAAGACGTGTG-3′/5′CATGGAAA CCAGAACACGTGG-3′, RPL13 forw/rev; 5′-CCTGGAGGAGAAGAGGAAAGAGA 3′/5′TTGAGGACCTCTGTGTATTTGTCAA-3′; TMPRSS11B forw/rev; 5′-CATTATGTA CAGGCACGGCAT3′/5′GTAAGTCTTCTCAACTGCCAG-3′.

### 4.4. Western Blotting

Total cell lysates were prepared using 2xLaemmli buffer, separated by SDS-PAGE for Western blot analysis as described before [61,62]. The target proteins were visualized and analyzed using the ChemiDoc™ MP Imaging System (Bio-Rad). The density of the bands was estimated using Image Lab™ software 6.1 (Bio-Rad). The relative expression of investigated proteins was calculated by normalizing the band intensities of the target protein to those of the housekeeping protein HSP90. SLC16A1 antibody (mouse, sc-365501, Santa Cruz) and TMPRSS11B antibody (rabbit, PA5-31481, Thermo-Fisher, Dreieich, Germany) were 1:200 and 1:500 diluted, respectively, in 5% skimmed milk in TBS-Tween (TBST), BSG and HSP90 antibodies (rabbit, #13257 and #4874, respectively, Cell Signaling, Frankfurt, Germany) were 1:1000 and 1:400 diluted, respectively, in 5% BSA in TBST. HRP-conjugated anti-mouse and rabbit antibodies (#7076 and #7074, respectively, Cell Signaling) were diluted 1:1000 in 5% skimmed milk in TBST.

### 4.5. Gene Knock-Down and Gene Silencing

The shRNA vector against TMPRSS11B (sc-89090-SH, Santa Cruz) or a control vector (sc-108060, Santa Cruz) was stably transfected in BxPC3 and Panc1 cells using Effectene reagent (Qiagen, Hilden, Germany) and subsequent culture in selection media (RPMI1640, 10% FCS, 1 mM NaPyr, 2 mM Glutamine) containing 1.0 and 1.5 µg/mL puromycine, respectively. For transient gene silencing, cells grown in 12-well plates were treated with 150 ng/mL SLC16A1 siRNA, BSG siRNA or control siRNA (no.SI03246614, SI00313733 and SI1027281, respectively, all Qiagen) using the *HiperFect* reagent (Qiagen).

### 4.6. Cloning and Stable Tranfection of TMPRSS11B cDNA

The coding region of TMPRSS11B (Genbank acc. no. NM_182502) was amplified from cDNA of Panc1 cells by conventional PCR using long-run Taq Polymerase (Thermo-Fisher) and the primers forward: 5′-CACCATGTACAGGCACGGCATATC-3′ and reverse: 5′GAGTCCAGTCTTGGATGTAATCC-3. The obtained amplicon was directly cloned into the pcDNA3.1-*Topo*-V5/His vector (Thermo-Fisher) and the obtained expression vector was validated by DNA sequence analysis (Genewiz/Azenta, Leipzig Germany). T3M4 cells were stably transfected with the TMPRSS11B expression vector or a control pcDNA3.1 plasmid using RPMI1640, 10% FCS, 1 mM NaPyr, 2 mM Glutamine containing 400 µg/mL G418 as the selection medium.

### 4.7. Colony Formation Assay

Cells were seeded in 6-well plates (500 cells/well) and cultured for 7–10 days. Then, cells were washed twice with PBS, fixed with methanol/acetic acid (3:1) for 5 min and stained with 0.1% (*w*/*v*) crystal violet. Plates were photographed using the Chemidoc-XRS^TM^transiluminator (BioRad). Colonies >0.25 mm diameter were counted and plating efficiency was calculated as the ratio of colony number/cells initially seeded.

### 4.8. Fluorometric iLACCO1 Lactate Uptake Assay

Cells seeded on 12-well culture plates were transfected with GFP-based lactate sensor iLACCO1 vector [63] kindly provided by Dr. Robert Campbell (Quebec, Canada) or the lactate-insensitive control vector (ΔiLACCO1) using the Effectene reagent (Qiagen). Then, cells were incubated with lactate uptake buffer (10 mM Hepes, 1 mM MgCl_2_, pH 7.6) for 1 h. Then, plates were measured in a microplate fluorescence reader (Tecan, Infiplex2) at 488 nm to detect basal fluorescence. Afterwards, lactate was added at a final concentration of 10 mM and fluorescence was recorded at 30 sec intervals until 2 min followed by measurement at 1 min intervals until 10 min. Fluorescence intensity differences between each time point (*Ft*) and basal fluorescence (*Fo*) were calculated and the ratio ((*Ft*-*Fo*)/*Fo*) was calculated. For comparative analyses the (*Ft*-*Fo*)/*Fo* ratios after 2 and 10 min were used.

### 4.9. Propidium Iodide Staining

After trypsinization, cells were washed twice in cold PBS containing 5 mM EDTA (PBSE) and then resuspended in 500 µL PBSE. For fixation, 500 µL chilled EtOH was added dropwise and the mixture was incubated at room temperature for 30 min. Fixed cells were collected by centrifugation, resuspended in 500 µL PBSE, incubated with 20 µg RNaseA for 30 min at room temperature and subsequently stained with propidium iodide (PI) by adding 500 µL of a 200 mg/mL PI stock solution. Samples were stored at 4 °C in the dark until counting using a FACSVerse cytometer (Becton Dickinson, Franklin Lakes, NJ, USA).

### 4.10. Measurement of Caspase-3/7 Activity

Caspase-3/7 activity was measured making use of the Caspase-Glo^®^ assay (Promega, Mannheim, Germany) according to the manufacturer’s instructions and as described [61]. Samples were measured in duplicates by fluorometry (Infinite M Plex, Tecan, Männedorf, Switzerland) and the resulting values were normalized to the respective protein concentration.

### 4.11. Patients and Tissues

Pancreatic tissues were obtained from patients during surgery [64]. Tissue specimens had been fixed in formalin and embedded in paraffin (FFPE). Hematoxylin and eosin-stained tissue sections had been assessed by board-certified surgical pathologists of the Department of Pathology, UKSH Campus Kiel. Only PDAC patients with a histologically confirmed Union for International Cancer Control (UICC) stage IIb (T3, tumor size > 4 cm; N1, spread to ≤ 3 lymph nodes; M0, no spread to distant sites) were included in the study.

### 4.12. Immunohistochemistry and Evaluation of Immunostaining

Three µm serial sections of FFPE tumor tissues from 31 PDAC patients were used. Deparaffinization of tissue sections was performed by incubating sections two times in xylene for 10 min. Afterwards, samples were rehydrated by applying a descending alcohol series, simultaneously blocking endogenous peroxidases by adding 1.5% (*v*/*v*) H2O2. Then, tissue sections were washed for 10 min with PBS before antigen retrieval was performed by incubating the sections in a microwave oven for 20 min in pre-warmed antigen retrieval buffer (citrate, pH 6.0 used for SLC16A1, BSG and KLF4, or Tris-EDTA, pH 9.0 for TMPRSS11B). Unspecific binding sites were blocked by incubation in PBS supplemented with 0.3% (*v*/*v*) Triton X-100 (PBS-T) and 4% (*w*/*v*) BSA for 1 h at RT. Immunostaining was carried out overnight at 4 °C using SLC16A1 antibody (mouse, SAB2702323, Sigma, Taufkirchen, Germany) at 1:150 dilution, BSG antibody (rabbit, #13257, Cell Signaling) at 1:250 dilution, TMPRSS11B antibody (rabbit, PA5-31481, Thermo-Fisher) at 1:100 dilution and KLF4 antibody (rabbit, PA5-23184, Thermo-Fisher) at 1:100 dilution in PBS-T plus 1% BSA. Immunostaining was visualized using anti-mouse or anti-rabbit peroxidase conjugates (*HRP Boost* mouse/rabbit, Cell Signaling) for 30 min at room temperature. Then, sections were washed three times in phosphate-buffered saline followed by peroxidase substrate reaction using the *AEC peroxidase* substrate kit (Abcam, Cambridge, UK) according to the manufacturer’s instructions. Mayer’s Haemalaun served as the counterstain (AppliChem, Darmstadt, Germany). Respective isotype controls were used to verify staining specificity, revealing no or only weak staining. Finally, immunostainings were evaluated using an Axio Imager M2 microscope (Zeiss, Jena, Germany) and 4 different fields at 40× magnification were analyzed. Using the following scoring, staining was evaluated: 0 = no evidence of staining; 1 = moderate staining (proportion <50%); 2 = moderate (proportion >50%); 3 = strong staining (proportion <50%); 4 = strong (proportion >50%).

### 4.13. Statistical Analysis

As indicated in the figure legends, normally distributed data were evaluated by two-tailed Student’s *t*-test (Excel 2021 Software; Microsoft-Windows 11) assuming equal variance and by ANOVA (GraphPad Prism 10.3.1) analysis of three groups (combined siRNA experiments). Non-parametric data were evaluated by the Wilcoxon–Mann–Whitney test. All data were included in statistical analysis with no randomization or blinding. No data points were excluded. *p* < 0.05 was considered statistically significant.

## 5. Conclusions

TMPRSS11B expression differentially impacts lactate transport in PDAC cells. In addition to its known promoting effect on lactate export through SLC16A1 and -A3 in highly glycolytic tumor cells, favoring Warburg metabolism and proliferation, it prevents lactate uptake by SLC16A1 in tumor cells, driving reverse Warburg metabolism. Accordingly, the absence of TMPRSS11B promotes the reverse Warburg metabolism in a subset of PDAC cells yielding essential malignant traits including chemoresistance and cancer stemness. Therefore, TMPRSS11B and its context-dependent role in cancer cell metabolism could be predictive for the prognosis of PDAC patients and the outcome of its therapeutic targeting.

## Data Availability

No new data were created or analyzed in this study. Data sharing is not applicable to this article.

## Supplementary Materials

The following supporting information can be downloaded at: https://www.mdpi.com/article/10.3390/ijms26115398/s1.

## Abbreviations

AEBSF, 4-(2-Aminoethyl)-benzolsulfonylfluoridhydrochlorid; BSG, Basigin; KLF4, Krüppel-like factor 4; LG, low-glucose medium; LGL, low-glucose medium plus lactate; NM, normal medium; Oct4, Octamer-binding transcription factor 4; PDAC, pancreatic adenocarcinoma; SLC16A1, solute carrier family member 16 A1; Sox2, sex-determining region Y (SRY)- box-2; TMPRSS11B, transmembrane protease serine 11B.
