# Supplementary material for: Transmembrane Protease Serine 11B Modulates Lactate Transport Through SLC16A1 in Pancreatic Ductal Adenocarcinoma—A Functional Link to Phenotype Heterogeneity
Source: Int J Mol Sci. 2025 Jun 4;26(11):5398. doi: 10.3390/ijms26115398 (PMC12155430; doi:10.3390/ijms26115398)
Supplement: Supplementary file 1 [file ijms-26-05398-s001.zip › ijms-3656054-supplementary.pdf]

**A)**	***G1***	***S***	***G2/M***
Panc1(co shR)/LG	54.68 ± 4.11	29.34 ± 2.33	15.98 ± 2.87
Panc1(TMP shR)/LG	55.32 ± 4.68	29.20 ± 1.74	15.47 ± 2.51
Panc1(co shR)/LGL	54.24 ± 3.96	29.31 ± 2.50	16.45 ± 3.12
Panc1(TMP shR)/LGL	53.84 ± 4.29	30.10 ± 2.72	16.06 ± 2.83
BxPc3(co shR)/LG	61.23 ± 5.42	21.14 ± 2.16	17.63 ± 3.05
BxPc3(TMP shR)/LG	60.50 ± 6.01	21.27 ± 2.00	18.23 ± 2.84
BxPc3(co shR)/LGL	57.31 ± 4.95 #	26.39 ± 2.39 #	16.30 ± 2.57
BxPc3(TMP shR)/LGL	55.27 ± 4.17	27.21 ± 2.24	17.52 ± 2.38
**B)**	***G1***	***S***	***G2/M***
T3M4(mock)/LG	58.77 ± 3.62	27.06 ± 2.57	14.17 ± 1.52
T3M4(TMP)/LG	59.46 ± 4.27	26.11 ± 2.28	14.43 ± 2.25
T3M4(mock)/LGL	54.46 ± 3.15	30.23 ± 2.95	15.31 ± 2.77
T3M4(TMP)/LGL	55.20 ± 4.51	29.65 ± 2.43	15.15 ± 2.41
**C)**	***G1***	***S***	***G2/M***
Panc1(co shR)/LG reseeded	56.78 ± 4.65	30.02 ± 2.44	13.20 ± 2.14
Panc1(TMP shR)/LG reseeded	57.05 ± 4.23	29.39 ± 1.89	13.56 ± 1.83
Panc1(co shR)/LGL reseeded	57.29 ± 4.03	33.01 ± 2.85	9.70 ± 2.92
Panc1(TMP shR)/LGL reseeded	53.04 ± 3.62*	36.73 ± 2.71*	10. 23 ± 2.57
BxPc3(co shR)/LG reseeded	52.69 ± 3.80	33.75 ± 2.40	13.56 ± 1.59
BxPc3(TMP shR)/LG reseeded	52.41 ± 4.13	32.92 ± 2.17	13.67 ± 1.72
BxPc3(co shR)/LGL reseeded	50.00 ± 3.56 #	37.04 ± 2.65 #	12.96 ± 1.53
BxPc3(TMP shR)/LGL reseeded	46.04 ± 3.15*	39.76 ± 2.88*	14.20 ± 1.90
**D)**	***G1***	***S***	***G2/M***
T3M4(mock)/LG	55.34 ± 2.78	30.12 ± 1.35	14.54 ± 1.22
T3M4(TMP)/LG	56.02 ± 3.34	30.01 ± 1.56	13.97 ± 1.05
T3M4(mock)/LGL	49.63 ± 2.75 #	33.58 ± 1.83 #	17.06 ± 1.64
T3M4(TMP)/LGL	54.72 ± 3.00*	29.50 ± 1.44*	15.78 ± 1.40
**E)**	***G1***	***S***	***G2/M***
Panc1(co shR)/control	56.48 ± 4.21	31.35 ± 2.07	12.17 ± 1.79
Panc1(TMP shR)/control	52.55 ± 3.35	34.70 ± 2.65	12.75 ± 1.94
Panc1(co shR)/SLC16A1	57.97 ± 4.38 *	29.83 ± 1.95	12.20 ± 1.76
Panc1(TMP shR)/SLC16A1	57.42 ± 3.81	28.79 ± 2.18 *	13.79 ± 2.15
Panc1(co shR)/BSG	58.10 ± 5.01	29.21 ± 2.24	12.69 ± 1.88
Panc1(TMP shR)/BSG	57.36 ± 4.72 *	28.31 ± 2.17 *	14.33 ± 2.21
BxPc3(co shR)/control	50.93 ± 2.82	33.66 ± 2.51	15.41 ± 1.74
BxPc3(TMP shR)/control	47.28 ± 2.48	36.02 ± 2.80	16.70 ± 2.11
BxPc3(co shR)/SLC16A1	53.35 ± 3.27	31.80 ± 2.44	14.85 ± 1.45
BxPc3(TMP shR)/SLC16A1	52.63 ± 2.70 *	32.64 ± 2.63	14.73 ± 1.66
BxPc3(co shR)/BSG	52.44 ± 3.33	32.91 ± 2.51	14.65 ± 1.83
BxPc3(TMP shR)/BSG	52.02 ± 2.99 *	32.63 ± 2.26	15.35 ± 2.31
**F)**	***G1***	***S***	***G2/M***
T3M4(mock)/control	50.22 ± 3.37	34.01 ± 2.74	15.77 ± 1.53
T3M4(TMP)/control	54.51 ± 3.75	30.15 ± 1.79	15.34 ± 1.36
T3M4(mock)/SLC16A1	55.40 ± 4.23 *	29.57 ± 1.52 *	15.03 ± 1.38
T3M4(TMP)/SLC16A1	54.98 ± 4.51	30.03 ± 1.88	14.99 ± 1.61
T3M4(mock)/BSG	56.02 ± 4.44 *	30.01 ± 1.70 *	13.97 ± 1.05
T3M4(TMP)/BSG	55.34 ± 4.20	30.52 ± 1.46	14.14 ± 1.22

Table S1. The effect of TMPRSS11B expression on the cell cycle of PDAC cells under reverse Warburg conditions. The PDAC lines Panc1 and BxPc3 transfected with TMPRSS11B (TMP shR) or control (co shR) shRNA and T3M4 cells stably transfected with an expression vector for TMPRSS11B cDNA (TMP) or the empty vector (mock) were cultured in low glucose (0.5g/L) medium without (LG) or with 20 mM lactate (LGL) for 48 h. Then, cells were either **A)** & **B)** directly submitted to PI staining and cell cycle analysis or **C)** & **D)** reseeded in normal medium (NM) for 24h followed by PI staining and cell cycle analysis. **E)** Panc1 and BxPc3 cells transfected with TMPRSS11B shRNA (TMP shR) or control shRNA (co shR) or **F)** T3M4

cells overexpressing TMPRSS11B (TMP) or not (mock) were first treated with control, SLC16A1 or BSG siRNA for 24 h followed by culture in low glucose medium plus 20 mM lactate (LGL) for 48 h. Then, cells were reseeded in NM for 24h followed by PI staining and cell cycle analysis. All data represent the mean ± SD from 4 independent experiments. Statistical significances are indicated as follows: **A)** and **C)**, *p<0.05 compared to “co shR/LGL” and #p<0.05 compared to “co shR/LG”; **D)**, *p<0.05 compared to “mock/LGL” and #p<0.05 compared to “mock/LG”; **E),** p<0.05 to „TMP shR/control siRNA“; **F)**, *p<0.05 to „mock/control siRNA“.

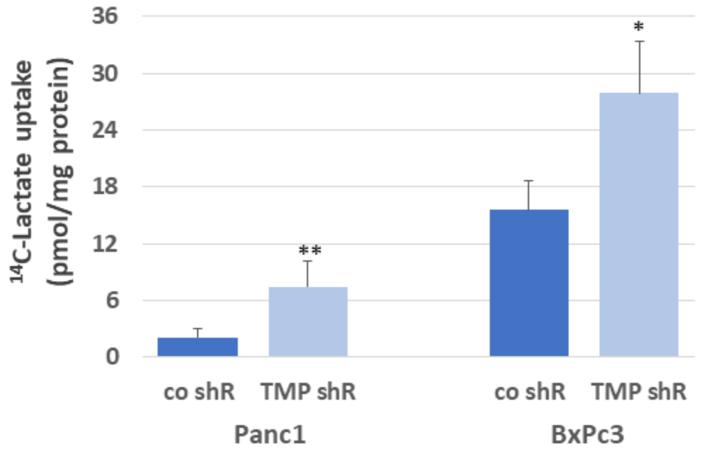

Figure S1. Effect of TMPRSS11B knock-down on ^{14}C-lactate uptake in Panc1 and BxPc3 cells. Panc1 and BxPc3 stably transfected with TMPRSS11B shRNA (TMP shR) or a control shRNA (co shR) were grown in 12-well plates. Then, the medium was replaced by 10 mM HEPES/pH 7.50, 5 mM KCl, 100 mM NaCl and 1 mM MgCl2 (uptake buffer) containing 2 µCi (0.5 µM) ^{14}C-L-lactate (American Radiolabeled Chemicals, Inc. ARC 0593) alone (= total) or together with 10 mM unlabelled lactate (to detect unspecific binding) and cells were incubated for 1–3 h at 37 °C. Afterwards, cells were washed 3× with ice-cold phosphate buffered saline and then lysed in 500 µl uptake buffer with 2% (w/v) sodium dodecyl sulphate. Lysates were submitted to liquid-scintillation beta-counting (LS-6500 instrument, Beckman Coulter, Krefeld, Germany). In parallel, protein concentrations in lysates from unlabelled cells were measured (DC Assay, BioRad) and used for normalization of beta-counting rates (triplicate measurements). Specific ^{14}C-lactate uptake was calculated by subtracting the normalized counting rates (total minus unspecific). Data show the specific incorporation of ^{14}C-lactate normalized to the amount of protein. Mean values ± SD from three independent experiments are shown. *p<0,05 & **p<0,001 compared to co shR.

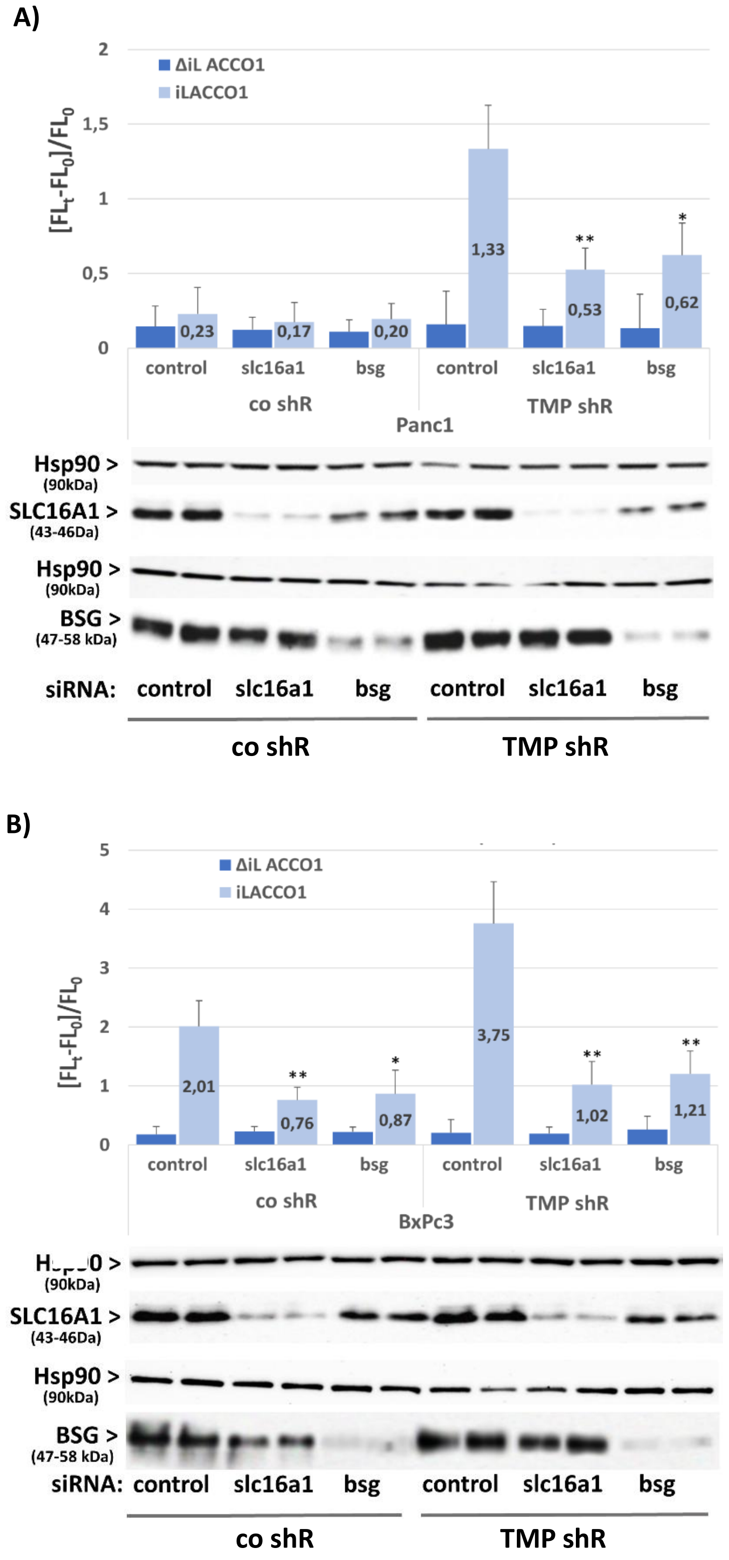

Figure S2. A) Panc1 and **B)** BxPc3 stably transfected with TMPRSS11B shRNA (TMP shR) or a control shRNA (co shR) were treated with control, SLC16A1 (slc16a1) or BSG (bsg) siRNA for 24h, followed by ΔiLACCO1 or iLACCO1 transfection and lactate uptake measurement 24h later (see Figure 2G). Afterwards, the cells were washed with PBS, lysed in SDS-PAGE sample buffer and prepared for western blot analysis with BSG and SLC16A1 antibodies. Western blot data show a representative from three independent experiments using Hsp90 as loading control.

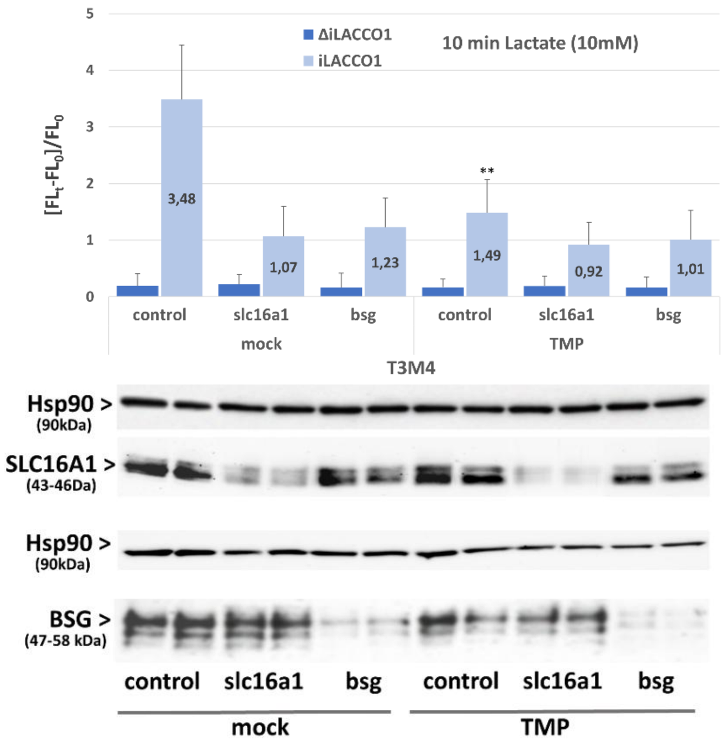

Figure S3. Stably mock or TMPRSS11B (TMP) transfected T3M4 cells were treated with control, SLC16A1 (slc16a1) or BSG (bsg) siRNA for 24h, followed by ΔiLACCO1 or iLACCO1 transfection and lactate uptake measurement 24h later (see Figure 3D). Afterwards, the cells were washed with PBS, lysed in SDS-PAGE sample buffer and prepared for western blot analysis with BSG and SLC16A1 antibodies. Western blot data show a representative from three independent experiments using Hsp90 as loading control.

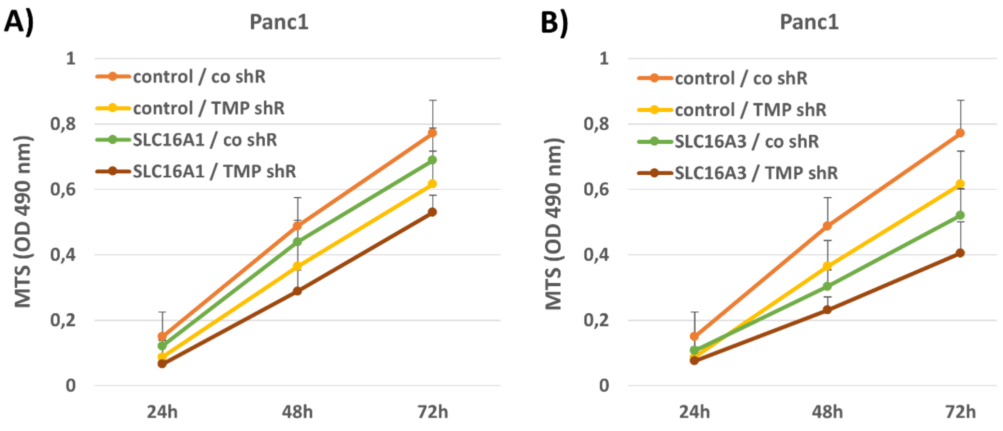

Figure S4. SLC16A1 and SLC16A3 dependency of the effect of TMPRSS11B expression on the growth of Panc1 cells. The PDAC cell line Panc1 stably transfected with TMPRSS11B (TMP shR) or control (co shR) shRNA was treated **A)** with control siRNA or SLCA16A1 siRNA or **B)** with control siRNA or SLCA16A3 siRNA followed by culture in normal medium containing 2g/L glucose. After the indicated periods, MTS assays (CellTiter 96®AQueous; Promega) were performed following the manufacturer's instructions. Viable cell numbers indicated by MTS optical density at 490 nm were analyzed using the *Infinite M Plex* instrument (Tecan). Data represent the mean ± SD of three independent experiments.
